# Dataset of autobiographical and future-related thoughts recorded during a laboratory vigilance task with working memory load manipulation

**DOI:** 10.1016/j.dib.2024.110831

**Published:** 2024-08-10

**Authors:** Krystian Barzykowski, Ewa Ilczuk, Lia Kvavilashvili

**Affiliations:** a Applied Memory Research Laboratory, Institute of Psychology, Faculty of Philosophy, Jagiellonian University, Kraków, Poland; b University of Hertfordshire, Hatfield, UK

**Keywords:** Mind wandering, Involuntary autobiographical memories, Involuntary future thoughts, Task-related thoughts, Task-unrelated thoughts, Vigilance task, Spontaneous cognition

## Abstract

The dataset provided in this article comprises frequencies of task-related thoughts, task-unrelated thoughts, involuntary autobiographical memories (IAMs), and involuntary future thoughts (IFTs) reported by adult participants during a laboratory vigilance task. Participants completed a vigilance task that included incidental cue words intended to trigger IAMs and IFTs, whose frequency was measured using random thought probes. The data were collected from two studies (*n* = 240 per study) in which working memory load and cue-presentation were manipulated. In both studies, participants completed an unexpected cue-recognition task after completing the vigilance task, which allowed for gathering additional data about noticing and remembering specific categories of cues (positive, neutral or negative). The dataset includes not only the frequencies of specific categories of thoughts but also data from numerous follow-up questions related to how participants perceived their performance in the task, such as their concentration level or perceived task difficulty. In conclusion the dataset contains three categories of variables: (1) variables related to participants and the conditions of the experimental sessions (i.e., age, gender, working memory load condition, etc.); (2) variables related to control questions (i.e., perceived task difficulty, emotional states, fatigue, etc.); and (3) variables related to performance in the vigilance task and the occurrence of thoughts (i.e., number of task-unrelated thoughts, number of involuntary memories, percentage of successfully recognized cues, etc.). This dataset could be reused to investigate many interesting relationships between cognitively engaging computer task characteristics and various parameters of task performance. Additionally, it could be used to conduct alternative or replication analyses to gain a deeper understanding of the relationship between working memory load and the experience of involuntary thoughts.

Specifications Table

**Table:** 

Subject	Experimental and cognitive psychology
Specific subject area	spontaneous thoughts, spontaneous cognition, involuntary past and future thoughts, mind wandering, task-unrelated thoughts
Type of data	Table; Raw, processed, filtered
Data collection	The data were collected from two studies (*n* = 240 per study). Participants completed a vigilance task while being exposed to verbal phrases, some of which could incidentally trigger task-unrelated thoughts, (incl. involuntary future thoughts and involuntary autobiographical memories). Throughout the task they were probed at random intervals to capture their thoughts. Additionally, the study manipulated working memory load. All data were collected using a software based on the Unity Real-Time Development Platform. Instructions and questions were displayed via the software on the subject's screen. The dataset does not include participants’ thoughts due to their qualitative nature. Instead, these thoughts underwent several stages of coding by expert judges to identify relevant categories.
Data source location	Jagiellonian University, Krakow, Poland
Data accessibility	All data are attached to the article. Repository name: RODBUK Data identification number: https://doi.org/10.57903/UJ/UXMKX6 Direct URL to data: https://uj.rodbuk.pl/dataset.xhtml?persistentId=doi:10.57903/UJ/UXMKX6
Related research article	

## 1 Value of the Data

• This dataset was obtained using a well-controlled and standardized experimental procedure; it provides valuable insights into the frequency and intensity of task-related and task-unrelated thoughts (including involuntary autobiographical memories and involuntary future thoughts) under different cognitive load conditions. Additionally, the data's utility for comprehensive analyses is enhanced by the inclusion of data from numerous follow-up questions assessing various psychological constructs, such as mood (PANAS), mental fatigue, and task difficulty.
• Researchers investigating mind wandering and spontaneous cognition, especially regarding task-related and task-unrelated thoughts as well as involuntary autobiographical memories and future thoughts, stand to benefit significantly from this dataset.
• This dataset allows for a wide variety of uses. For instance, it could be used to conduct alternative or replication analyses to gain a deeper understanding of the relationship between working memory load and the experience of involuntary thoughts. The data could also be utilized for meta-analyses related to studies of mind wandering in general, and spontaneous cognition in particular. The presence of control groups without any additional cognitive load (i.e., n-back task) could be also useful for investigating the experience of such thoughts in general.
• The data from the many control questions displayed during the procedure could also be used to draw conclusions on a meta-level regarding, for example, experimental conditions and participants’ reactions in general. For example, the data on the declared focus level, which was measured 23 times during the procedure, make it possible to analyze the temporal variability of concentration levels in relation to cognitive load or other variables. Additionally, data on the emotional valence of the cues may be valuable for analyzing how people remember (recognize) specific categories of phrases.

## 2 Background

The presented dataset is the outcome of a meticulously designed, well-controlled and standardized experimental procedure conducted in laboratory settings, coupled with a rigorous categorization process of non-quantitative data (see also [2]). Its publication provides a valuable avenue to further exploration of the cognitive mechanisms that underlie the occurrence of involuntary thoughts, as well as the performance of cognitively demanding tasks in general. This dataset adds value to the published research [1] by offering comprehensive insights into the nuanced relationship between cognitive load and spontaneous cognition, thereby enriching our understanding of human cognition.

## 3 Data Description

This dataset includes two separate files. The first file Batch#1-Study-1_Data_in_Brief_v3, contains the frequency of experiencing specific types of thoughts in Study 1, where working memory load (control, low, high) and cue presentation (simultaneous, sequential) were manipulated. The second file, Batch#1-Study-2_Data_in_Brief_v4, contains information from Study 2, where working memory load (control, low, high) and the type of working memory load (verbal, visuo-spatial) were manipulated. Table 1 provides descriptions of the variables in the datafile related to Study 1, while Table 2 provides descriptions related to Study 2.

**Table 1:** Study 1: Descriptions of variables.

Variable	Description
*Variables related to participants and the conditions of the experimental sessions:*	
ID	Participants’ individual ID numbers.
gender	Gender of participants. Possible options: male, female, other.
age	Age of participants (calculated based on birth date).
load_vs_control_condition	Information about working memory load in the condition performed by participants. Possible options: Control (control group without any additional n-back task); WM_Load (experimental group with an additional n-back task – n-back difficulty level not specified).
n_back_difficulty	Precisely defined working memory load condition performed by participants. Possible options: Control (group with no additional n-back task); 1-back (experimental group with low working memory load); 3-back (experimental group with high working memory load).
letters_simultaneous_vs_sequential_cue	Information about the cue presentation. Possible options: Simultaneous (cues presented on the same slides as working memory task stimuli); Sequential (cues presented on separate slides).
Load_condition_after_exclusion	Working memory load condition after excluding participants who had less than 50 % correct answers in the n-back task or guessed the true purpose of the study (collecting involuntary autobiographical memories and future thoughts).
Cue_presentation_after_exclusion	Information about the cue presentation condition after excluding participants who had less than 50 % correct answers in the n-back task or guessed the true purpose of the study (collecting involuntary autobiographical memories and future thoughts).
*Variables related to control questions:*	
PANAS_positive_before	Score on the PANAS positive affect scale performed before the vigilance task (scale: 1–5). *1 - Very Slightly or Not at All* *2 - A Little* *3 - Moderately* *4 - Quite a Lot* *5 - Extremely*
PANAS_negative_before	Score on the PANAS negative affect scale performed before the vigilance task (scale: 1–5). *1 - Very Slightly or Not at All* *2 - A Little* *3 - Moderately* *4 - Quite a Lot* *5 - Extremely*
physical_fatigue_before	Score on the “To what extent are you physically fatigued at this very moment?” question that was displayed before the vigilance task (scale: 1–7)*.
mental_fatigue_before	Score on the “To what extent are you mentally fatigued at this very moment?” question that was displayed before the vigilance task (scale: 1–7)*.
PANAS_positive_after	Score on the PANAS positive affect scale displayed after the vigilance task (scale: 1–5, as described above).
PANAS_negative_after	Score on the PANAS negative affect scale displayed after the vigilance task (scale: 1–5, as described above).
physical_fatigue_after	Score on the “To what extent are you physically fatigued at this very moment?” question that was displayed after the vigilance task (scale: 1–7).*
mental_fatigue_after	Score on the “To what extent are you mentally fatigued at this very moment?” question that was displayed after the vigilance task (scale: 1–7).*
task_concentration_general	Score on the “To what extent have you been concentrating on the task?” question (scale: 1–7).*
vertical_lines_concentration	Score on the “To what extent have you been concentrating on the vertical lines?” question (scale: 1–7).*
phrases_concentration	Score on the “To what extent have you been concentrating on the phrases on the screen?” question (scale: 1–7).*
task_performing_well	Score on the “To what extent was it important to perform the task as well as you could?” question (scale: 1–7).*
task_difficulty	Score on the “To what extent was the task difficult?” question (scale: 1–7).*
task_interesting	Score on the “To what extent was the task interesting?” question (scale: 1–7).*
cues_interfering	Score on the “How much did the verbal phrases interfere with the vigilance task?” question (scale: 1–7).*
thoughts_suppressing	Score on the “How much did you suppress involuntary thoughts?” question (scale: 1–7).*
cues_ignoring	Score on the “How much did you ignore the verbal phrases?” question (scale: 1–7).*
*Variables related to performance in the vigilance task and the occurrence of thoughts:*	
vigilance_targets_detected	Proportion of detected vigilance targets (vertical lines) out of total vigilance targets.
vigilance_targets_detection_RT	Mean reaction time (in seconds) of correct reactions to vigilance task targets (vertical lines).
concentration_partial_rates_mean	Mean concentration level reported by participants during each of the 23 thought probe stops.
concentration_stop1 *(…23)*	Score on the “To what extent were you concentrating just before the program stopped?” question that was shown each time the program stopped (23 times in total). (scale: 1–7).*
correct_nback_response	Proportion of detected n-back targets out of the total number of n-back targets presented during the task.
correct_nback_response_RT	Mean reaction time (in seconds) for correct responses to n-back targets during the task.
cue_recognition	Percentage of incidental verbal cues that were successfully recognized at the end of the vigilance task.
cue_recognition_positive	Percentage of positive cue words that were successfully recognized at the end of the vigilance task.
cue_recognition_neutral	Percentage of neutral cue words that were successfully recognized at the end of the vigilance task.
cue_recognition_negative	Percentage of negative cue words that were successfully recognized at the end of the vigilance task.
attention_checks	Proportion of correct responses to attention checks: Participants were instructed to press a specific number key on their computer keyboard (e.g., “3” in response to a question about their concentration on the vigilance task during the thought probe). The proportion of correct responses reflects adherence to these instructions.
task_unrelated_thoughts	Number of involuntary task-unrelated thoughts.
task_related_thoughts	Number of involuntary task-related thoughts.
involuntary_autobiographical_memories	Number of involuntary autobiographical memories experienced during the study.
involuntary_future_thoughts	Number of involuntary future thoughts experienced during the study.

⁎ In general, response labels were created according to the following template: 1 – corresponds to not endorsing the item at all; 4 – corresponds to medium endorsement; 7 – corresponds to strong endorsement. For example, the scale for physical fatigue was as follows: “To what extent are you physically fatigued at this very moment?”; 1 = *I* am not physically fatigued at all, 2 = *I* am not physically fatigued, 3 = *I* am slightly physically fatigued, 4 = *I* am somewhat physically fatigued, 5 = *I* am rather physically fatigued, 6 = *I* am physically fatigued, 7 = *I* am very physically fatigued. All points on the scale were clearly labeled.

**Table 2:** Study 2: Descriptions of variables.

Variable	Description
*Variables related to participants and the conditions of the experimental sessions:*	
ID	Participants’ individual ID numbers.
gender	Gender of participants. Possible options: male, female, other.
age	Age of participants (calculated based on birth date).
load_vs_control_condition	Information about working memory load in the condition performed by participants. Possible options: Control (control group without any additional n-back task); WM_Load (experimental group with an additional n-back task – n-back difficulty level not specified).
n_back_difficulty	Precisely defined working memory load condition performed by participants. Possible options: Control (group with no additional n-back task); 1-back (experimental group with low working memory load); 3-back (experimental group with high working memory load).
type_n-back	Information about the type of the n-back task condition. Possible options: Verbal (participants react whenever the letter displayed on the screen matches the letter presented *n* trials earlier); Spatial (participants react whenever the position of the letter-square displayed on the screen corresponds to the location presented *n* positions prior).
Load_condition_after_exclusion	Working memory load condition after excluding participants who had less than 50 % correct answers in the n-back task or guessed the true purpose of the study (collecting involuntary autobiographical memories and future thoughts).
Type_nback_after_exclusion	Information about the type of the n-back task condition after excluding participants who had less than 50 % correct answers in the n-back task or guessed the true purpose of the study (collecting involuntary autobiographical memories and future thoughts).
*Variables related to control questions:*	
PANAS_positive_before	Score on the PANAS positive affect scale performed before the vigilance task (scale: 1–5). *1 - Very Slightly or Not at All* *2 - A Little* *3 - Moderately* *4 - Quite a Lot* *5 - Extremely*
PANAS_negative_before	Score on the PANAS negative affect scale performed before the vigilance task (scale: 1–5). *1 - Very Slightly or Not at All* *2 - A Little* *3 - Moderately* *4 - Quite a Lot* *5 - Extremely*
physical_fatigue_before	Score on the “To what extent are you physically fatigued at this very moment?” question that was displayed before the vigilance task (scale: 1–7)*.
mental_fatigue_before	Score on the “To what extent are you mentally fatigued at this very moment?” question that was displayed before the vigilance task (scale: 1–7)*.
PANAS_positive_after	Score on the PANAS positive affect scale displayed after the vigilance task (scale: 1–5, as described above).
PANAS_negative_after	Score on the PANAS negative affect scale displayed after the vigilance task (scale: 1–5, as described above).
physical_fatigue_after	Score on the “To what extent are you physically fatigued at this very moment?” question displayed after the vigilance task (scale: 1–7).*
mental_fatigue_after	Score on the “To what extent are you mentally fatigued at this very moment?” question displayed after the vigilance task (scale: 1–7).*
task_concentration_general	Score on the “To what extent have you been concentrating on the task?” question (scale: 1–7).*
vertical_lines_concentration	Score on the “To what extent have you been concentrating on the vertical lines?” question (scale: 1–7).*
phrases_concentration	Score on the “To what extent have you been concentrating on the phrases on the screen?” question (scale: 1–7).*
task_performing_well	Score on the “To what extent was it important to perform the task as well as you could?” question (scale: 1–7).*
task_difficulty	Score on the “To what extent was the task difficult?” question (scale: 1–7).*
task_interesting	Score on the “To what extent was the task interesting?” question (scale: 1–7).*
cues_interfering	Score on the “How much did the verbal phrases interfere with the vigilance task?” question (scale: 1–7).*
thoughts_suppressing	Score on the “How much did you suppress involuntary thoughts?” question (scale: 1–7).*
cues_ignoring	Score on the “How much did you ignore the verbal phrases?” question (scale: 1–7).*
*Variables related to performance in the vigilance task and thoughts occurrence:*	
vigilance_targets_detected	Proportion of detected vigilance targets (vertical lines) out of total vigilance targets.
vigilance_targets_detection_RT	Mean reaction time (in seconds) of correct reactions to vigilance task targets (vertical lines).
concentration_partial_rates_mean	Mean concentration level declared by participants during each of the 23 thought probe stops.
Concentration_stop1 *(…23)*	Score on the “To what extent were you concentrating just before the program stopped?” question that was shown each time the program stopped (23 times in total; scale: 1–7).*
correct_nback_response	Proportion of detected n-back targets out of the total number of n-back targets presented during the task.
correct_nback_response_RT	Mean reaction time (in seconds) for correct responses to n-back targets during the task.
cue_recognition	Percentage of successfully recognized cues displayed during the vigilance task.
cue_recognition_positive	Percentage of successfully recognized positive cues displayed during the vigilance task.
cue_recognition_neutral	Percentage of successfully recognized neutral cues displayed during the vigilance task.
cue_recognition_negative	Percentage of successfully recognized negative cues displayed during the vigilance task.
attention_checks	Proportion of correct responses to attention checks: Participants were instructed to press a specific number key on their computer keyboard (e.g., “3” in response to a question about their concentration on the vigilance task during the thought probe). The proportion of correct responses reflects adherence to these instructions.
task_unrelated_thoughts	Number of involuntary task-unrelated thoughts.
task_related_thoughts	Number of involuntary task-related thoughts.
involuntary_autobiographical_memories	Number of involuntary autobiographical memories experienced during the study.
involuntary_future_thoughts	Number of involuntary future thoughts experienced during the study.

⁎ In general, response labels were created according to the following template: 1 – corresponds to not endorsing the item at all; 4 – corresponds to medium endorsement; 7 – corresponds to strong endorsement of the item. For example, the scale for physical fatigue was as follows: “To what extent are you physically fatigued at this very moment?”; 1 = *I* am not physically fatigued at all; 2 = *I* am not physically fatigued; 3 = *I* am slightly physically fatigued; 4 = *I* am somewhat physically fatigued; 5 = *I* am rather physically fatigued; 6 = *I* am physically fatigued, 7 = *I* am very physically fatigued. All points on the scale were clearly labeled.

## 4 Experimental Design, Materials and Methods

### 4.1 Participants

480 participants (240 in Study 1 and 240 in Study 2) were recruited via social media platforms to participate in the experimental session in the laboratory; they received a modest reward of PLN 50 (c.a., $13). During the recruitment phase, participants were not informed about the investigation into spontaneous thoughts regarding past and future events. Instead, the study was presented as an examination of “attentional focus” to prevent participants from intentionally retrieving such thoughts during task performance [3,4]. After exclusions as described above, there were 450 participants in total: 225 in Study 1 and 225 in Study 2. In Study 1, there were 167 females; 51 males and 7 participants choose the “other” option; Mean age = 22.8, SD = 4.07; 5 participants did not indicate their age. In Study 2, there were 173 females, 44 males, and 6 participants choose the “other” option; Mean age = 22.6, SD = 5.12; 1 participant did not indicate his age.

### 4.2 Procedure and materials

Both studies were conducted in a laboratory setting to ensure controlled and standardized conditions and maximize participants’ concentration. Participants were tested in groups ranging from two to eleven individuals per laboratory session. They received a brief overview of the study before providing consent. Subsequently, at individual computer stations they completed a computer-based procedure comprising several components: the Positive and Negative Affect Schedule (PANAS), which was administered before and after the vigilance task; the vigilance task (including a concurrent n-back task); a cue-recognition task; additional control questions, and a phase for describing and categorizing thoughts. Importantly, Study 1 and Study 2 had a very similar design but differed in the experimental manipulation. In Study 1, working memory load (control, low, high) and cue presentation method (simultaneous, sequential) were manipulated, while Study 2 manipulated working memory load (control, low, high) and its type (verbal, visuo-spatial). Despite these differences, the procedural elements remained largely consistent across both studies.

### 4.3 PANAS and additional control questions

To minimize potential intergroup variations, we administered the Positive and Negative Affect Schedule (PANAS, [5]), comprising 30 items aimed at assessing the intensity of participants’ current negative (15 items) and positive (15 items) emotional states. On a 5-point Likert scale, participants rated the extent to which the provided adjectives reflected their emotional condition. To score the PANAS 30-item scale, ratings for the respective items for Positive Affect and Negative Affect are summed. The reliability coefficients (internal consistency and stability) of the Polish version of PANAS range from 0.73 to 0.95 [5].

Additionally, participants were also asked about their physical and mental fatigue, responding on a 7-point scale. Both PANAS and the questions regarding physical and mental fatigue were administered before and after the vigilance task in both Study 1 and Study 2. These additional control items, previously used in studies (e.g. [6,7,8,9]), have proven useful in measuring background variables.

### 4.4 Vigilance task with additional n-back task

**Fig. 1:**
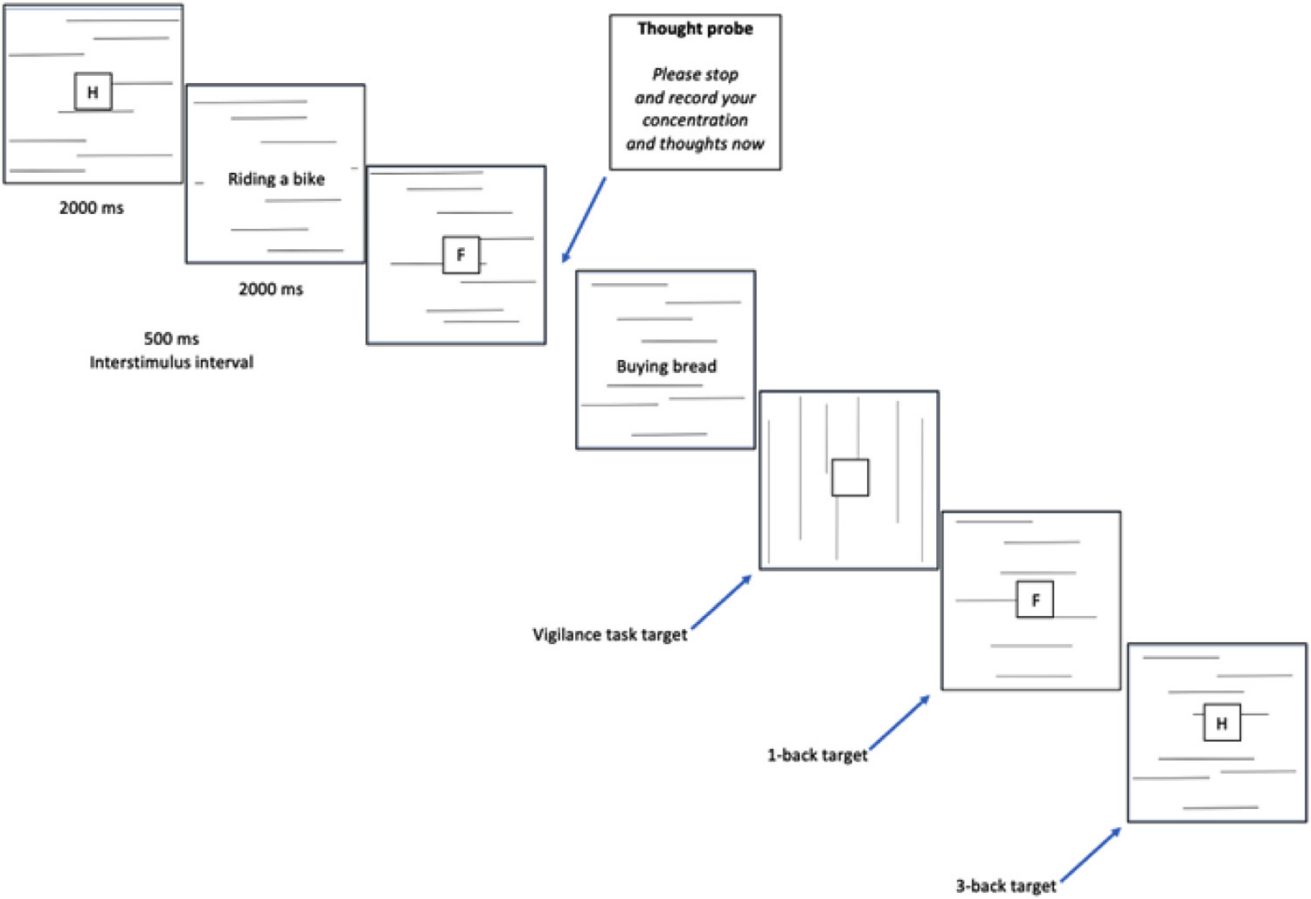
Vigilance task with verbal working memory load and sequential presentation of cues, as employed in both Study 1 and Study 2.

In both studies, participants completed a computerized version of the vigilance task procedure [2], concurrently with an additional n-back task. During the vigilance task, participants were tasked with detecting rarely occurring (15 in total) target slides featuring vertical lines among a larger set of infrequent non-target slides displaying horizontal lines (785 slides in total) (see Fig. 1). Each slide was presented for 2 s, occasionally accompanied by short verbal phrases (e.g., “riding a bike”) at the center. A pool of 270 phrases drawn from previous investigations and comprising neutral, positive, and negative content was evenly distributed across the slides. Throughout the task, participants were probed 23 times at random intervals to rate their level of concentration on a 7-point scale and to record their thoughts at the moment of the thought probe, indicating whether the recorded thought had occurred spontaneously or deliberately. Instructions for recording thoughts were provided: “Write down briefly, in a few words, what is on your mind. It does not matter at all whether you find it interesting or not. Everything is important, regardless of what it is about. If, for some reason, you do not want to describe the content of your thoughts, write an X or describe them more generally. Use this, however, only on very rare occasions”. It is important to note that the basic version of the vigilance task described above is a monotonous and simple task, and it was performed by the control groups in both studies.

For the manipulation of cognitive load in both studies, an additional n-back task was presented to participants during the vigilance task: verbal in Study 1, and verbal vs. visuo-spatial in Study 2.

**Fig. 2:**
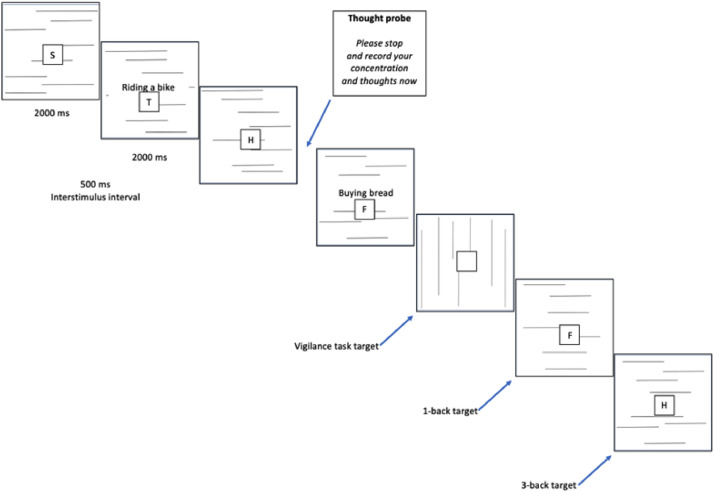
Vigilance task with verbal working memory load and simultaneous cue presentation, as employed in Study 1.

**In Study 1** (see also Fig. 1, Fig. 2), participants were presented with a sequence of uppercase consonants (B, C, D, F, G, H, K, M, N, P, R, S, T, W, Z) individually, displayed at the center of the screen for 2 s each, with a 0.5-s interval between them. Participants in the experimental groups were tasked with detecting vertical lines and were additionally instructed to respond by pressing a green button (“X” on the keyboard) whenever the letter displayed on the screen matched the letter presented either 1 trial earlier (under low working memory load conditions) or 3 trials earlier (under high working memory load conditions). They had 2.5 s to respond to the target with a permissible maximum reaction time (RT) of 2.5 s and a minimum RT of 100 ms. Premature or late responses were not recorded and were classified as misses. Participants in the control group underwent the same procedure but were not instructed to respond in any way to the displayed letters. In addition, in Study 1 the presentation of incidental verbal cues was manipulated. Half of the participants had cues displayed simultaneously with the stimuli associated with the n-back task, where the word phrase and the letter square were present on the same slide (see Fig. 2). The other half of participants had cues displayed separately from the stimuli associated with the n-back task, where the word phrase could not be displayed on the same slide as the letter square (see Fig. 1).

**Fig. 3:**
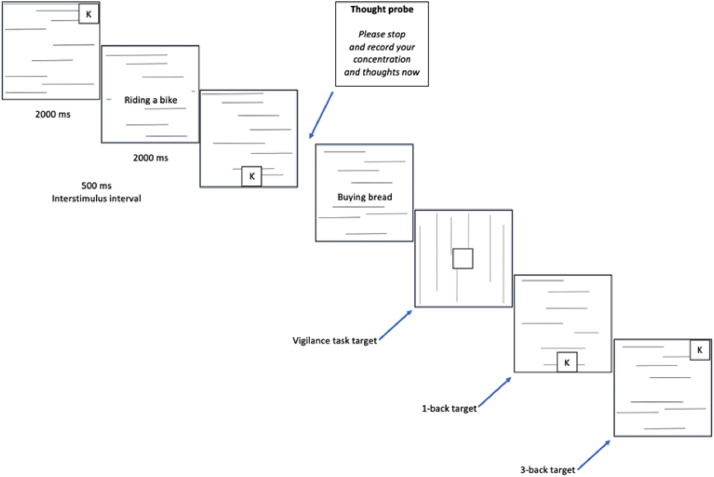
Vigilance task with visuo-spatial working memory load and sequential cue presentation, as employed in Study 2.

**In Study 2** (see also Fig. 1 and Fig. 3), half of the participants were assigned to perform a verbal n-back task similar to that in Study 1, while the other half completed a visuo-spatial n-back task. The spatial n-back task mirrored the parameters of the verbal task used in Study 1, with each slide displayed for 2 s with a 0.5-s interstimulus interval, etc.. However, stimuli were presented at one of nine distinct locations on the computer screen (see Figure 3). Participants were explicitly instructed to react whenever the position of the letter-square displayed on the screen corresponded either to the immediately preceding location (under low working memory load conditions) or to the location presented three positions prior (under high working memory load conditions) in the sequence. Finally, in Study 2, cue presentation was not manipulated. All participants had cues displayed sequentially on separate slides.

### 4.5 Cue-recognition task

In both studies, participants completed an unexpected cue-recognition task immediately after the vigilance task. A total of 84 cues were presented, with 42 cues randomly sampled from the cue pool utilized during the vigilance task. The remaining 42 cues were novel selections from a broader pool of 800 cues used in previous studies on spontaneous past and future thoughts (e.g., [3]). Importantly, each set of old and new cue phrases comprised an equal distribution of neutral, positive, and negative phrases (*N* = 14 each). These cues were presented in two predetermined pseudo-random sequences, counterbalanced across participant groups and experimental conditions. Participants responded to each cue displayed on the screen in a self-paced manner, without imposed time constraints.

### 4.6 Summary of study 1 and study 2 flow

In **Study 1**, conducted between May and June 2022, working memory load and cue presentation were manipulated. Participants were randomly assigned to three groups: control (without additional n-back task), low working memory load (additional 1-back verbal task) and high working memory load (additional 3-back verbal task). Cues were presented either together with working memory task stimuli on the same slide or on separate slides (sequential vs. simultaneous condition). In **Study 2**, conducted between October 2022 and January 2023, participants were also randomly assigned to three groups: control (without additional n-back task), low working memory load (additional 1-back verbal task) and high working memory load (additional 3-back verbal task). Cues were presented sequentially for all participants. Additionally, the type of working memory load was manipulated, with half of the participants performing a verbal n-back task and the other half performing a visuo-spatial n-back task.

In both studies, before and after the vigilance task, participants completed PANAS. They were also given an unexpected cue-recognition task after the vigilance task, where they had to determine whether the presented cues were displayed (YES/NO) during the vigilance task. Participants in both studies elaborated on and classified their thoughts, and additional control questions were used.

### 4.7 Control questions

The control questions used in the study were aimed at assessing various aspects of participants’ experiences and perceptions during the experimental tasks. These questions included:

(1) An open-ended question regarding participants’ perceptions of the true goal of the study.
(2) Ratings (on a 7-point scale) of the task's perceived (a) fatigue, (b) attention demand, (c) difficulty, (d) interest.
(3) Ratings (on a 7-point scale) of participants’ concentration levels on (a) the task in general, (b) the verbal phrases, (c) the vertical lines, (d) the square with letters.
(4) Ratings (on a 7-point scale) of the importance of performing the computer task well;
(5) Ratings (on a 7-point scale) of the extent to which (a) involuntary thoughts, (b) verbal phrases and (c) square-letters were experienced as interfering.
(6) Ratings (on a 7-point scale) of the extent to which participants suppressed involuntary thoughts.
(7) Ratings (on a 7-point scale) of the extent to which participants ignored verbal phrases.

### 4.8 Description of thoughts and the categorization phase

In both studies, following the completion of the vigilance task, cue recognition, and control questions, participants were prompted to describe and categorize the thoughts they had previously written. Initially, participants received concise instructions outlining the nature of autobiographical memories and future thoughts. Subsequently, participants reviewed each thought they had recorded during the vigilance task, proceeding in the same order as they had been written. They were then instructed to classify each thought as an autobiographical memory, future-oriented thought, thought relating to the current situation, or another type of thought by selecting the appropriately labeled button. Additionally, participants provided a more detailed description of each thought.

### 4.9 Categorization process of involuntary past and future thoughts

The data do not include the content of the thoughts written by the study participants due to their qualitative nature. Instead, they contain information on the numbers of specific categories of thoughts experienced by participants, including task-unrelated thoughts, task-related thoughts, involuntary autobiographical memories, and involuntary future thoughts. For this reason, a description of the thought categorization process will be briefly provided (for a detailed description, see [2]). This process consisted of four stages and utilized classifications from both the participants and the competent judges’ assessments.

(1) The goal of the first stage of the thought categorization process was to distinguish between task-related and task-unrelated thoughts. As described in [2, p. 9], task-related thoughts were defined as those clearly connected to the ongoing task, reflecting full engagement with the ‘*here and now*,’ and directly related to the task at hand (e.g., ‘*I'm waiting for the vertical lines*,’ ‘*I pressed the red button*’). In contrast, task-unrelated thoughts were those not related to the task or the current situation. Specifically, these thoughts did not reference the vigilance task and could pertain to the past (e.g., ‘*romantic dinner at a cozy restaurant by the beach with my boyfriend last summer*’), the present (e.g., ‘*feeling cold in the room*’), or the future (e.g., ‘*upcoming exam I have in a few weeks during the examination session*’).
(2) Following extensive training, two competent judges coded all recorded thoughts into these categories utilizing both short and long descriptions of the thoughts obtained from the vigilance task and the subsequent thoughts description phase.
(3) In the second stage, task-unrelated thoughts were automatically categorized as deliberate or involuntary, based on the participants’ own classifications.
(4) During the third stage, involuntary task-unrelated thoughts classified by participants as related to the past or future underwent screening by a competent judge to identify any obvious mistakes in the participants’ decisions, employing a double-check procedure. The judge was aware of the category to which the thought was assigned by participants.
(5) In the fourth stage, involuntary task-unrelated thoughts classified by participants as “other” or “current situation” were re-categorized by competent judges using a control-check procedure. The judges were unaware of the participants’ original classifications. This stage was necessary because memories and thoughts about the future sometimes fall into these categories (“other” and “current situation”), and without this re-categorization, they would not be counted. As discussed in [2, p. 9], participants often choose the ‘*other*’ category for atemporal thoughts (e.g., “*Do dwarfs exist?*”) or when unsure how to classify their thoughts, especially if they span different time frames (e.g., “*I thought I pressed the button correctly and also thought about the lunch I will have after the study*”). Similarly, participants might select the ‘*current situation*’ category even when the thought is only minimally relevant to the present moment (e.g., “*I'm sad because I was reminded of yesterday's conflict*”). This can lead to the misclassification of past and future thoughts. To ensure accuracy, we had two trained judges re-code the thought descriptions labeled as ‘*other*’ or ‘*current situation*’ by the participants. The judges categorized the thoughts into the following groups: (a) memories: thoughts about the past, including events that happened, were experienced, or were witnessed; (b) future thoughts: thoughts regarding the future, such as wishes, plans, tasks, or upcoming events; and (c) other: thoughts that do not fit into the categories of memories or future thoughts. Importantly, the judges were instructed to always assign the category ‘other’ when in doubt.
(6) Moreover, as part of the data preparation, a research assistant reviewed all responses to the question about the true purpose of the study and identified those who guessed that the purpose was to detect autobiographical memories and thoughts about the future.
(7) As described in [2, p. 9], any discrepancies or disagreements between judges were resolved through discussion and consensus to ensure the accuracy and reliability of the final data. Importantly, to ensure the consistency of judgments, the inter-rater reliability was assessed prior to resolution by calculating Cohen's Kappa coefficient. The judges achieved excellent inter-rater reliability at this final coding stage (Cohen's Kappa = 0.84), indicating robust consistency in the coding process.

As a result, in Study 1 we obtained 2517 involuntary task-unrelated thoughts (iTUTs), 1590 task-related thoughts (TRTs), 907 involuntary autobiographical memories (IAMs) and 804 involuntary future thoughts (IFTs); in Study 2, we obtained 2657 iTUTs, 1524 TRTs, 1238 IAMs, and 671 IFTs. The number of thoughts was then processed through appropriate statistical analyses (e.g., in Study 1: two-way ANOVAs with working memory load group (none, low, high) and cue-presentation (simultaneous, sequential) as between subject variables. In Study 2: two-way ANOVAs with the type of working memory load (verbal vs. spatial) and working memory load (none, low, high).

## Limitations

Given that the thought numbers are based on the categorization of competent judges, the use of the data must be limited to the categories of interest to the data presented here. Additionally, the research sample consisted largely of healthy students, which may make it difficult to generalize conclusions from analyses made on the data to different sub-populations (e.g., older participants). One could also argue that the probe-caught method may not be as sensitive in detecting the effects of working memory load as the self-caught method, where participants stop themselves to report the occurrence of spontaneous thoughts. Participants in the working memory load condition could have experienced an increased frequency of IFTs and IAMs in the time periods between consecutive stops. Ideally, future studies could employ self-caught methods to study the effects of working memory load and cue presentation.

## Ethics Statement

The University Research Ethics Committee approved the study (no: KE/39_2021). Written consent for participation was obtained prior to data collection. Participants were informed that they were free to withdraw from the study at any point. Study 1 and Study 2 were conducted in accordance with the University guidelines for safe working during the COVID-19 pandemic.

## Declaration of Generative AI and AI-Assisted Technologies in the Writing Process

During the preparation of this work, Krystian Barzykowski used ChatGPT 3.5 to proofread sentences and enhance the language and readability. After using this tool, the author reviewed and edited the content as needed and takes full responsibility for the content of the publication.

## CRediT Author Statement

**Krystian Barzykowski**: Conceptualization, Methodology, Validation, Resources, Data curation, Writing – review & editing, Visualization, Supervision, Funding acquisition. **Ewa Ilczuk**: Conceptualization, Methodology, Investigation, Writing – original draft, Writing – review & editing, Project administration. **Lia Kvavilashvili:** Conceptualization, Methodology, Writing - Review & Editing.

## Data Availability

Dataset of autobiographical and future-related thoughts recorded during laboratory vigilance task with working memory load manipulation (Original data) (RODBUK).
